# Acute Severe Immune Thrombocytopenia Following Dupilumab Use for Eustachian Tube Dysfunction: A Case Report

**DOI:** 10.7759/cureus.87724

**Published:** 2025-07-11

**Authors:** Amanda J Lu, Sean Morris, Lisa Weissmann

**Affiliations:** 1 Medicine, Brigham and Women's Hospital, Harvard Medical School, Boston, USA; 2 Medicine, Mount Auburn Hospital, Harvard Medical School, Cambridge, USA

**Keywords:** drug-induced immune thrombocytopenia, dupilumab, : eustachian tube dysfunction, mucocutaneous bleeding, t-helper

## Abstract

Immune thrombocytopenic purpura (ITP) is a rare hematologic disorder characterized by isolated thrombocytopenia and mucocutaneous bleeding. While drug-induced ITP (DITP) is recognized with certain medications, reports associated with dupilumab, a monoclonal antibody used for atopic dermatitis and other inflammatory conditions, are exceedingly rare. We present a case of acute, severe thrombocytopenia occurring two weeks after dupilumab initiation for eustachian tube dysfunction in a 76-year-old male. Comprehensive evaluation excluded secondary causes, supporting a diagnosis of dupilumab-induced ITP. Treatment with corticosteroids and intravenous immunoglobulin led to rapid platelet recovery. Given the expanding use of dupilumab, awareness of this rare but potentially serious adverse event is crucial. Clinicians should remain vigilant for mucocutaneous bleeding in patients receiving biologic agents and initiate appropriate treatment promptly. Moreover, enhanced pharmacovigilance and further research are needed to elucidate the incidence, risk factors, and mechanisms of dupilumab-associated hematologic toxicity.

## Introduction

Immune thrombocytopenic purpura (ITP) is an uncommon hematologic disorder characterized by isolated thrombocytopenia and an increased risk of mucocutaneous bleeding. It may present as a primary condition or secondary to underlying causes, such as infections, autoimmune diseases, malignancies, or medications [1,2]. Current clinical guidelines define ITP as a platelet count below 100 × 10⁹/L in the absence of other identifiable causes of thrombocytopenia [3]. Diagnosis is primarily clinical and requires exclusion of secondary etiologies. In cases of drug-induced immune thrombocytopenia (DITP), hallmark features include abrupt onset of severe thrombocytopenia (often <20 × 10⁹/L), temporal correlation with drug exposure, resolution upon drug discontinuation, and recurrence upon re-exposure [4]. While drug-dependent platelet antibody testing may assist diagnosis, it is not universally accessible or definitive [5].

Dupilumab, a monoclonal antibody targeting the interleukin-4 receptor alpha subunit, is approved for the treatment of moderate-to-severe atopic dermatitis, asthma, and chronic rhinosinusitis with nasal polyposis [6]. It has also demonstrated efficacy in improving eustachian tube dysfunction (ETD) and otologic symptoms in patients with aspirin-exacerbated respiratory disease (AERD) [7]. Although generally well tolerated, rare hematologic adverse events such as delayed-onset thrombocytopenia and immune pancytopenia consistent with Evans syndrome have been reported [8,9]. Clinical trial data indicate that thrombocytopenia of any grade occurs in approximately 0.2-0.3% of patients, with Grade ≥3 events occurring in less than 1% [10]. Real-world pharmacovigilance data confirm that hematologic events are infrequent, and specific reports of thrombocytopenia remain uncommon [11]. To date, only two published cases have described probable dupilumab-induced ITP, highlighting the rarity of this complication [8,9].

Despite its low incidence, dupilumab-associated ITP is clinically significant given the potential severity of acute presentations. We report a case of acute, severe thrombocytopenia occurring two weeks after dupilumab initiation for ETD in a 76-year-old male, contributing to the limited but growing literature on this rare adverse event.

## Case presentation

A 76-year-old male with a medical history significant for relapsing-remitting multiple sclerosis, paroxysmal supraventricular tachycardia (PSVT), ETD with bilateral nasal polyps, hypertension, and prior thrombotic stroke presented with a three-day history of recurrent blood-filled oral bullae and petechiae on both lower extremities. He had initiated dupilumab therapy for ETD two weeks prior. The patient denied recent infections, vaccinations, or new medications and reported no use of alcohol, tobacco, or illicit drugs.

On examination, he was alert and oriented with stable neurological findings. Multiple blood-filled bullae (1.5-2 cm in diameter) were observed on the oral mucosa, along with petechiae on both lower extremities.

Diagnosis of ITP was made per the 2019 American Society of Hematology (ASH) guidelines, which define ITP as a platelet count below 100,000/μL in the absence of other causes [3]. Laboratory studies revealed severe thrombocytopenia with a platelet count of 2,000/μL (Table 1). Peripheral smear showed no schistocytes, and coagulation studies were within normal limits, effectively excluding disseminated intravascular coagulation (DIC). Hemolysis was ruled out by normal reticulocyte count, stable hemoglobin, normal bilirubin, and unremarkable coagulation parameters (Table 2), thereby excluding thrombotic thrombocytopenic purpura (TTP) [3,12]. Serologic testing for hepatitis B, hepatitis C, and HIV was negative (Table 3), eliminating infectious etiologies [13]. These comprehensive assessments support a diagnosis of ITP by excluding secondary causes, consistent with the ASH criteria.

**Table 1:** Trend analysis of complete blood count (CBC) results: pre- and post-treatment (days 0–3) PLTS: platelets; RBCs: red blood cells; WBCs: white blood cells

Variables	Reference	Day 0	Day 1 AM	Day 1 PM	Day 2 AM	Day 2 PM	Day 3 AM	Day 3 PM
WBC	4.00 x 10^3^u/L to 11.00 x 10^3^u/L	4.61	5.39	5.85	7.00	9.37	10.04	11.94
RBC	4.30 x 10^6^/uL to 5.80 x 10^6^/uL	4.46	4.44	4.23	4.31	4.37	4.40	4.51
Hemoglobin	13.5 g/dL to 17.5 g/dL	14.5	14.1	13.3	13.7	13.9	13.9	14.3
Hematocrit	41.0%-53.0%	42.5	42.0	39.6	41.3	41.3	41.2	42.7
PLTS	150 x 10*3u/l to 400 x 10^3^u/L	2	1	6	23	47	71	89

**Table 2:** Assessment of coagulation parameters: prothrombin time (PT), INR, and aPTT aPTT: activated partial thromboplastin time, INR: PR: prothrombin time, INR: international normalized ratio

Variables	Reference	Results
PT	10.0-13.2 s	12.50
INR	0.9-1.1	1.1
aPTT	28.0-40.0 s	35.4

**Table 3:** Serological and molecular testing for HBV, HCV, and HIV infections HBV: hepatitis B; HCV: hepatitis C

Variables	Reference	Results
HBS Ag	Negative	Negative
HBS Ab	≥ 10 mIU/mL	Negative
HCV Ab	Non-reactive	Non-reactive
HIV AG/AB	Negative	Negative

**Figure 1:**
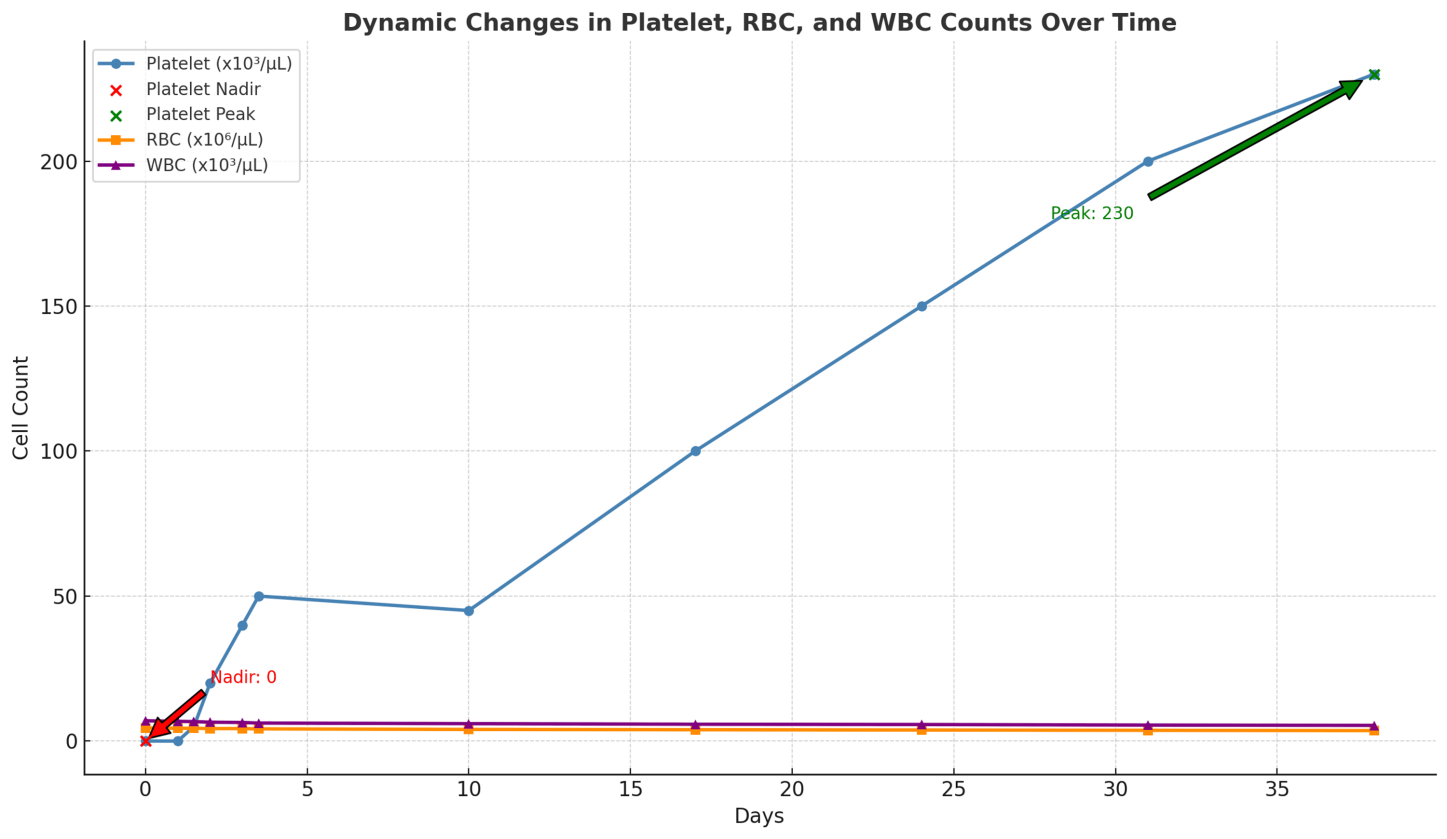
Dynamic changes in platelet, RBC, and WBC counts over time

The patient was treated with high-dose corticosteroids (dexamethasone 40 mg IV daily for three days) starting on day 0, along with a single dose of intravenous immunoglobulin (IVIG) at 0.4 g/kg on day one. Due to active buccal mucosal bleeding, one unit of platelets was transfused. Dupilumab was discontinued, and rivaroxaban was held, given the high risk of bleeding. The patient was monitored with twice-daily complete blood counts, showing a progressive rise in platelet counts to 23,000/μL on day one, 47,000/μL on day two, and 89,000/μL on day three. He was discharged on day three with a scheduled weekly outpatient follow-up, during which platelet counts continued to improve and red blood cell indices remained stable (Table 1; Figure 1). The timeline of dupilumab-induced thrombocytopenia onset, diagnosis, and treatment is summarized in Table 4.

**Table 4:** Timeline of dupilumab-induced thrombocytopenia onset, diagnosis, and treatment

Timeline	Events
Day -14	Dupilumab administered
Day -3	Blood-filled oral bullae and petechial rash on bilateral legs observed
Day 0	ED visit
	Platelet count = 1 x 10³/μL
	Dexamethasone 40 mg IV
Day 1	IVIG administered at 0.4 g/kg
	One unit of platelets transfused
	Platelet count = 6 x 10³/μL
Day 2	Dexamethasone 40 mg IV
	Platelet count = 47 x 10³/μL
Day 3	Dexamethasone 40 mg IV
	Platelet count = 89 x 10³/μL

## Discussion

Drug-induced thrombocytopenia is a recognized adverse effect of several medications, with immune-mediated platelet destruction triggered by agents such as heparin, quinine, and monoclonal antibodies, including rituximab and cetuximab [2,4]. Dupilumab-induced thrombocytopenia is exceedingly rare; one reported case described this complication occurring one year after initiation for atopic dermatitis, suggesting an immunologic mechanism involving Th2 cell modulation through IL-4/IL-13 inhibition, potentially triggering autoantibody production [8]. Another case reported immune pancytopenia consistent with Evans syndrome two months after dupilumab initiation [9].

In this case, the patient developed acute, severe isolated thrombocytopenia following dupilumab initiation, accompanied by mucocutaneous bleeding consistent with ITP. The temporal association between drug exposure and symptom onset supports a diagnosis of drug-induced ITP. The immunologic profile suggested a shift toward dominant Th1 responses characterized by IFN-γ production and cell-mediated immunity [14].

Management of drug-induced ITP typically involves discontinuation of the offending agent and administration of corticosteroids, IVIG, or other immunosuppressive therapies [3,4]. Alternative diagnoses, such as aplastic anemia or viral infections, were excluded by the absence of abnormalities in other cell lines, lack of viral symptoms, and negative infectious workup. The patient’s platelet count improved significantly following corticosteroid and IVIG treatment, further substantiating the diagnosis.

Given emerging reports of hematologic adverse events, including DITP, enhanced post-marketing surveillance of dupilumab is warranted. Establishing a multicenter pharmacovigilance registry would facilitate the systematic collection of real-world hematologic data, particularly in patients with autoimmune comorbidities, concurrent medications, or prolonged therapy. Retrospective analyses utilizing large health databases could help characterize incidence, temporal patterns, and risk factors associated with DITP in dupilumab-treated populations [1,2]. Integration with existing adverse event reporting systems, such as the FDA Adverse Event Reporting System (FAERS) and EudraVigilance, would strengthen safety monitoring and inform clinical guidelines [3,4].

The following are the differential diagnoses:

Bone marrow failure syndrome: It typically presents with pancytopenia and requires bone marrow biopsy. Normal hemoglobin and white blood cell counts make this unlikely [15].

Disseminated intravascular coagulation (DIC): It is characterized by bleeding and clotting with coagulopathy, but normal coagulation markers rule this out [12].

Infectious causes (HI and hepatitis B or C): Excluded by negative serologies [15].

Platelet sequestration (e.g., splenomegaly, liver disease): History and labs unremarkable.

Malignancy (leukemia, lymphoma): The lack of systemic symptoms such as fever, weight loss, or lymphadenopathy makes this unlikely [16].

## Conclusions

Dupilumab-associated ITP is an exceptionally rare but potentially serious adverse event warranting clinical vigilance, especially as the drug’s use broadens. This case underscores the importance of recognizing the temporal relationship between dupilumab initiation and acute severe thrombocytopenia consistent with drug-induced ITP. A comprehensive diagnostic evaluation to exclude alternative causes and prompt treatment with corticosteroids and IVIG can lead to favorable outcomes. Given the rarity of such events, ongoing pharmacovigilance through dedicated registries and large-scale data analyses is essential to elucidate the incidence, risk factors, and mechanisms of dupilumab-induced hematologic toxicity. Early recognition and management remain critical to patient safety and informed therapeutic decision-making.
